# Molecular Barriers to Zoonotic Transmission of Prions

**DOI:** 10.3201/eid2001.130858

**Published:** 2014-01

**Authors:** Marcelo A. Barria, Aru Balachandran, Masanori Morita, Tetsuyuki Kitamoto, Rona Barron, Jean Manson, Richard Knight, James W. Ironside, Mark W. Head

**Affiliations:** The University of Edinburgh, Edinburgh, Scotland, UK (M.A. Barria, R. Knight, J.W. Ironside, M.W Head);; Canadian Food Inspection Agency, Ottawa, Ontario, Canada (A. Balachandran);; Japan Blood Products Organization, Kobe, Japan (M. Morita);; Tohoku University Graduate School of Medicine, Sendai, Japan (T. Kitamoto);; University of Edinburgh, Easter Bush, Scotland, UK (R. Barron, J. Manson)

**Keywords:** prion disease, Creutzfeldt-Jakob disease, bovine spongiform encephalopathy, scrapie, CJD, BSE, chronic wasting disease, in vitro assay, cell-free system, protein misfolding diseases, prions, zoonoses

## Abstract

The risks posed to human health by individual animal prion diseases cannot be determined a priori and are difficult to address empirically. The fundamental event in prion disease pathogenesis is thought to be the seeded conversion of normal prion protein to its pathologic isoform. We used a rapid molecular conversion assay (protein misfolding cyclic amplification) to test whether brain homogenates from specimens of classical bovine spongiform encephalopathy (BSE), atypical BSE (H-type BSE and L-type BSE), classical scrapie, atypical scrapie, and chronic wasting disease can convert normal human prion protein to the abnormal disease-associated form. None of the tested prion isolates from diseased animals were as efficient as classical BSE in converting human prion protein. However, in the case of chronic wasting disease, there was no absolute barrier to conversion of the human prion protein.

## Abstract 2

Chronic wasting disease in elk might be a threat to human health.

Prion diseases are rare fatal neurodegenerative conditions that affect humans and animals. The human diseases include Creutzfeldt-Jakob disease (CJD), Gerstmann-Sträussler-Scheinker disease, and fatal familial insomnia. Most cases of human prion disease are apparently spontaneously occurring (sporadic CJD [sCJD]) or are associated with mutations in the human prion protein gene, designated *PRNP* (genetic CJD, Gerstmann-Sträussler-Scheinker disease, or fatal familial insomnia). A small minority of cases are acquired by inadvertent human-to-human transmission during medical or surgical treatments (iatrogenic CJD).

In contrast, animal prion diseases are generally acquired. This applies to scrapie in sheep, transmissible mink encephalopathy, and chronic wasting disease (CWD) in deer and elk. No credible evidence exists of a link between scrapie and any human prion disease, despite the endemicity of scrapie in many parts of the world and the consequent likely human exposure to the scrapie agent, which has been attributed partly to a species barrier between sheep and humans. However, strong epidemiologic, pathologic, and molecular evidence does indicate that the epidemic of bovine spongiform encephalopathy (BSE), primarily in the United Kingdom during the 1980s, resulted in a zoonotic form of CJD termed variant CJD (vCJD). BSE/vCJD is the only known zoonotic prion disease strain.

After identification of BSE and vCJD, active surveillance for animal prion diseases in Europe and elsewhere has identified rare atypical prion diseases in sheep and cattle. These include Nor98 or atypical scrapie in sheep (*1*) and 2 prion diseases of cattle, bovine amyloidotic spongiform encephalopathy or L-type BSE (*2*) and H-type BSE (*3*), both of which have a pathology and epidemiology distinct from classical or C-type BSE (*4*). In addition to these new (or newly described) diseases of farmed sheep and cattle, CWD in cervids is an acquired, probably contagious disease that affects captive and free-ranging deer and elk populations primarily in North America (*5*).

Their distinctive epidemiology, clinical features, neuropathology, PrP biochemistry, and transmission characteristics suggest that scrapie, atypical scrapie, C-type BSE, H-type BSE, L-type BSE, and CWD represent distinct prion strains in their respective species (*6*,*7*). Within scrapie and CWD, natural strain variation also occurs. The prion hypothesis posits that the posttranslational conformational conversion of a host’s normal cellular prion protein (PrP^C^) by the abnormal form of the prion protein (PrP^Sc^) is the fundamental event in prion disease pathogenesis and that PrP^Sc^ itself constitutes the infectious agent. It follows that an aspect of prion host range may be a species barrier operating at the molecular level that depends on compatibility between the PrP^Sc^ from 1 species and the PrP^C^ from another. Similarities in the species-specific primary *PRNP* sequences may account for part of this effect, but prion strain and host *PRNP* polymorphic genotype, both of which probably find expression in the conformation of PrP, affect susceptibility in ways not yet fully understood.

A relatively simple empirical approach to assessing this molecular barrier is to use cell-free PrP conversion assay techniques (*8*,*9*) to determine the relative efficiency of PrP conversion using natural “seeds” from an infectious prion source from the brain of 1 species and a normal brain “substrate” from another species. We have previously reported the use of protein misfolding cyclic amplification (PMCA) as a model of cross-species prion transmission of C-type BSE in cattle and sheep to humans (*10*). Here we report a comparative study of the ability of sheep, cattle, and deer prions to convert normal human PrP in this same cell-free system.

## Methods

Ovine, bovine, and cervine frozen brain tissue from prion disease–affected and –unaffected animals were obtained by request from the Animal Health Veterinary Laboratory Agency TSE Archive (AHVLA, Weybridge, UK). The cases and brain regions supplied were selected on the basis of proven disease status and of brain region with an expected high PrP^Sc^ load, characteristic of the particular prion disease. The prion disease status of the animals involved was determined at AHVLA and/or the Canadian Food Inspection Agency’s Ottawa laboratory (Ottawa, ON, Canada) by neuropathology and PrP immunohistochemistry. The classical scrapie specimen was of brain stem from a field suspect of the animal prion protein gene *Prnp* ARQ/ARQ genotype, and a brain stem specimen from an unaffected scrapie suspect of the same genotype was also supplied. The atypical scrapie specimen was of parietal cortex, also from a field suspect, but of the ARQ/AHQ genotype, and a corresponding negative control animal specimen was also supplied. The C-type BSE samples were of brain stem from confirmed positive C-type BSE suspects obtained through passive surveillance, and the corresponding negative control specimen was similarly obtained. Both the H- and L-type BSE specimens were of frontal cortex from successful experimental bovine transmissions conducted at AHVLA. Mid-brain tissue from a confirmed CWD-positive and control-negative (unaffected) elk (both with *Prnp* codon 132MM genotype) was also supplied through the AHVLA.

Frozen half brains from inbred transgenic mouse lines expressing human PrP^C^ of the *PRNP* codon 129 methionine (129MM) and valine (129VV) genotypes (*11*–*13*) were used for PMCA substrate preparation. The production of PMCA substrates from stably transfected human 293F cells overexpressing human PrP^C^ (exogenous *PRNP* codon 129M and endogenous *PRNP* codon 129MM) has been described (*14*). A *PRNP* codon 129 valine expressing counterpart was engineered by suppressing expression of endogenous *PRNP* codon 129MM expression with RNAi and transient transfection with a *PRNP* codon 129 valine expression vector (designated 129V).

Human brain tissues (frontal cortex) were sampled from frozen half brains collected at autopsy with the appropriate consent for tissue retention and research use. The vCJD specimen was from a patient (*PRNP* codon 129MM) with definite vCJD as defined by established criteria. The non-CJD human brain specimens used for PMCA substrate preparation were frontal cortex from patients with Guillain-Barré syndrome (129MM) and dementia with Lewy bodies (129VV). sCJD specimens from patients with the MM1 and VV2 subtypes of the disease were used as reference standards in certain Western blotting experiments. Ethical approval for the use of these tissues in this study is covered by Local Research Ethics Committee 2000/4/157.

Brain homogenates were prepared by using a manual homogenizer and chilled conversion buffer (150 mM NaCl, 1% Triton X-100, 1X protease inhibitor cocktail in 1× phosphate-buffered saline) to obtain a final 10% wt/vol solution. The homogenized tissue was cleared by centrifugation at 2,000 rpm for 40 s in a refrigerated centrifuge (4°C), and the supernatant was aliquoted and stored at −80°C (*15*).

We prepared homogenates (10% wt/vol) of C-type BSE, scrapie, CWD, L-type BSE, H-type BSE atypical scrapie, and vCJD brain. We followed the same method used for the substrate brain homogenate.

PMCA experiments were carried out in PCR tubes. Aliquots of 10% substrate brain homogenate (or 20% cell extracts) were mixed with 10% prion disease brain seeds in a final volume of 120 μL. Low molecular weight heparin was included at 100 μg/mL in all PMCA reactions (*15*). Before sonication, 19 μL of the PMCA reaction mixture (termed “frozen” sample) was taken for comparison with the amplified sample (termed “sonicated” sample). The reactions were incubated into the microplate horn of a programmable sonicator (Misonix 4000, Misonix, Farmingdale, NY, USA) at 37°C. A total of 96 PMCA cycles were performed comprising 20 s of sonication (at an amplitude of 90%) followed by 29 min 40 s of incubation for every cycle (*16*).

Tissue homogenates and PMCA reaction products were digested with proteinase K (50 μg/mL for 1 h at 37°C) and detected by Western blotting (*10*). Detection was with 3F4 or 6H4 antibodies diluted in 1× phosphate-buffered saline, 0.05% Tween 20 (*10*). The monoclonal antibody 9A2 was obtained from Central Veterinary Institute Wageningen UR (Lelystad, the Netherlands) (*7*). Membranes were developed by using peroxidase conjugated secondary antibody and a luminescent peroxidase substrate ECL-Plus (*10*). Finally, blots were exposed to a photographic film and the image acquired using the XRS digital CCD camera system (Bio-Rad Laboratories, Hercules, CA, USA). The antibody 6H4 recognizes an epitope in the protease-resistant core of human PrP spanning amino acids 145–153, and it cross-reacts with bovine, ovine, and cervine PrP. 3F4 also reacts with a sequence in the protease resistant core of the human PrP spanning positions 106–112, but 3F4 does not recognize PrP from species other than humans and hamsters. The combination of protease-digestion and detection by 3F4 in these experiments therefore provides a very sensitive method for detecting newly formed human protease-resistant prion protein (PrP^res^) (*10*,*17*). The antibody 9A2 reacts with human PrP amino acids 99–101 and cross-reacts with ovine, bovine, and cervine PrP.

## Results

**Figure 1:**
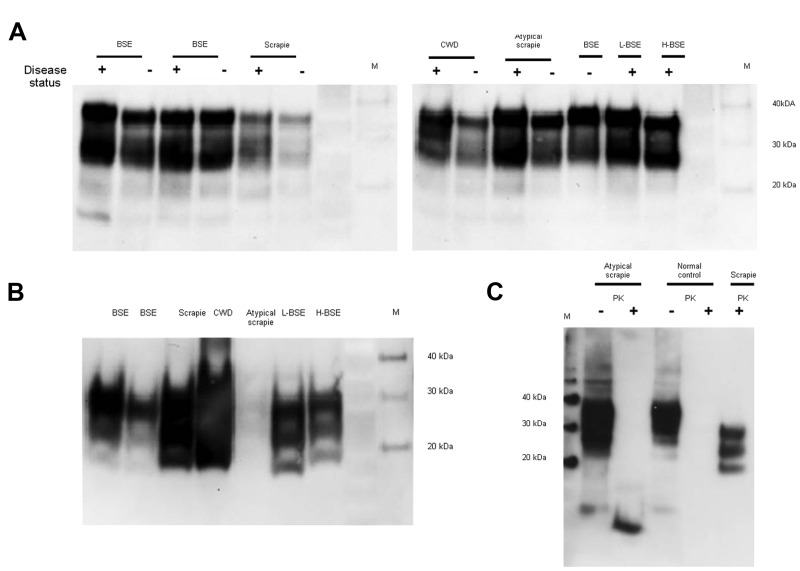
Determination of total PrP and PrP^res^ level in animal tissues. To characterize the PrP expression levels (total PrP), brain homogenates were analyzed by Western blot without digestion with PK. Nineteen microliters of each 10% wt/vol homogenate was loaded in each lane (A). To detect the PrP^res^ in the samples, PK digestion (50 μg/mL) was performed to remove PrP^C^, and the samples were then reanalyzed (B). The atypical scrapie and matched normal control animal samples were further analyzed by Western blot both with (+) and without (–) prior PK digestion (C) by comparing 3 μL of undigested homogenates with 100 μL of the PK-digested sample concentrated by centrifugation. Five microliters of a PK-digested classical scrapie brain homogenate was analyzed in parallel for comparison. The detection antibody was 6H4 in (A) and (B) and 9A2 in (C). PrP, prion protein; PrP^res^, protease-resistant PrP; PK, proteinase K; PrP^c^, normal cellular PrP; M, molecular marker; BSE, bovine spongiform encephalopathy; +, animal prion disease sample; –, matched normal animal control sample; CWD, chronic wasting disease; L-BSE, L-type BSE; H-BSE, H-type BSE.

PrP was confirmed in all animal brain samples in the form of 2 major bands in the 20–40-kDa molecular mass range, probably corresponding to full-length diglycosylated PrP (upper band) and N terminally truncated diglycosylated or full-length monoglycosylated PrP (lower band). The levels of PrP and the electrophoretic pattern were broadly similar between prion disease and corresponding negative control (unaffected) animal brain samples (Figure 1, panel A). However, proteinase K digestion showed differences in the amount of PrP^res^ contained in these samples. PrP^res^ was most abundant in classical scrapie and CWD samples; lower levels were seen in C-, H- and L-type BSE samples and were barely detectable at this level of sensitivity in atypical scrapie (Figure 1, panel B). Normalizing the Western blot PrP^res^ signal by adjusting sample loading volumes demonstrated the expected PrP^res^ relative mobilities and glycosylation types (data not shown). When larger volumes of brain homogenate were analyzed, a characteristic <10-kDa band was present in the atypical scrapie specimen (Figure 1, panel C).

**Figure 2:**
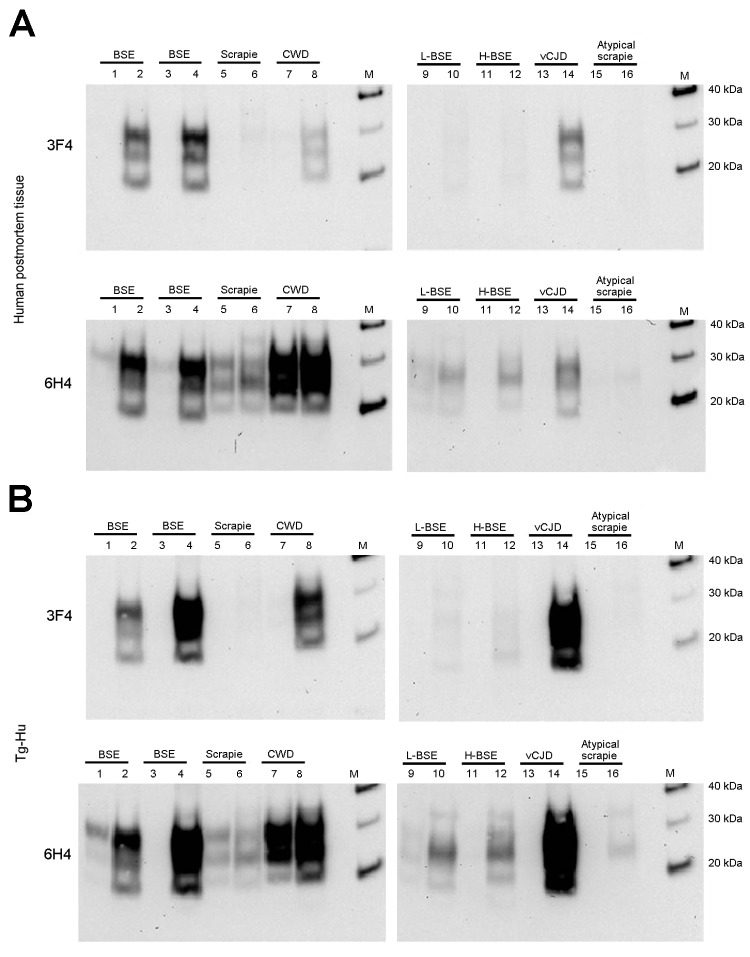
PMCA of *PRNP* codon 129MM human brain homogenate and humanized transgenic mice brain homogenate seeded with C-BSE, scrapie, CWD, L-BSE, H-BSE, vCJD, and atypical scrapie. PMCA reactions using *PRNP* 129MM human brain homogenate (human postmortem tissue) (A) and *PRNP* 129MM humanized transgenic mouse brain homogenate (Tg-Hu) (B) were seeded (1:3) with animal prion disease brain as indicated. Lanes 1, 3, 5, 7, 9, 11, 13, and 15 show the samples without PMCA. Samples in lanes 2, 4, 6, 8, 10, 12, and 14 were subjected to PMCA. Western blotting used the antibody 3F4 that enables the specific detection of human PrP. To compare the PrP^res^ levels into the seeds (before the PMCA), antibody 6H4 was also used. PMCA, protein misfolding cyclic amplification; BSE, bovine spongiform encephalopathy; CWD, chronic wasting disease; vCJD, variant Creutzfeldt-Jakob disease; PrP, prion protein; PrP^res^, protease-resistant PrP; M, molecular marker.

We evaluated the susceptibility of the human PrP (129MM) to in vitro conversion first using the human brain homogenate substrate. Western blotting with antibodies 3F4 and 6H4 both showed readily detectable amplification in the samples seeded with C-type BSE and vCJD. (Figure 2, panel A, lanes 2, 4, and 14, compared with lanes 1, 3, and 13). Scrapie, L-type BSE, H-type BSE, and atypical scrapie reactions did not show detectable human PrP^res^ formation with the 3F4 antibody (Figure 2, panel A, lanes 6, 10, 12, and 16). However, 3F4 detected human PrP^res^ in the reaction seeded with the CWD brain homogenate (Figure 2, panel A, lane 8). Humanized transgenic mouse (129MM) brain substrate similarly showed efficient amplification of vCJD and C-type BSE and readily detectable amplification of CWD PrP^res^ using 3F4 (Figure 2, panel B). Faint bands were seen in L-type BSE and H-type BSE PMCA reactions when the 6H4 antibody was used. These bands most likely represent conversion of endogenous bovine PrP^C^ from the inoculum converted to PrP^res^, rather than conversion of human PrP^C^ from the substrate (compare lanes 11 and 12 for 6H4 and 3F4 in Figure 2, panels A, B).

**Figure 3:**
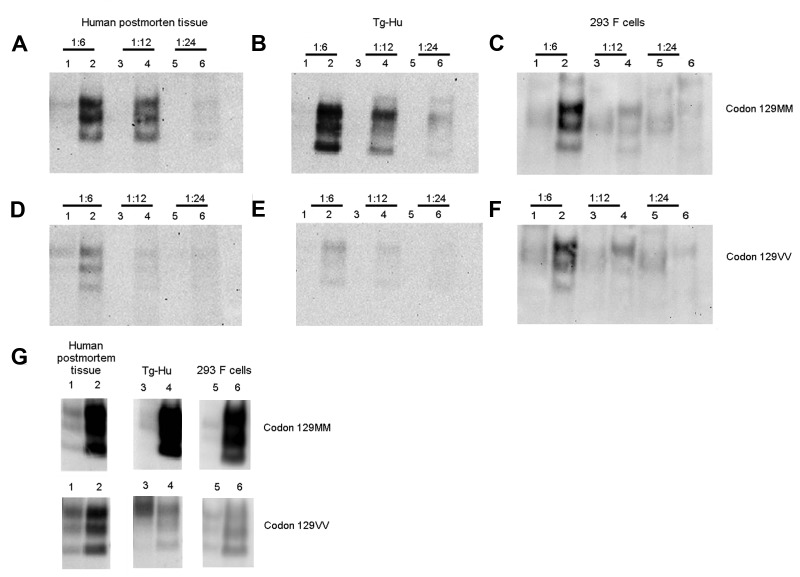
Susceptibility of human brain, humanized transgenic mice brain, and 293F cell extract PrP^C^ to in vitro conversion by CWD brain homogenate. PMCA seeded with serial dilutions of CWD brain homogenate (1:6, 1:12, and 1:24) were mixed with substrate from 3 different sources: human brain homogenate (A, D); transgenic mice that express the human PrP (B, E); and 293F human cell extract (C, F). The substrates contained human PrP 129M (A–C) or PrP 129V (D–F). These same substrates were seeded with vCJD brain homogenate at 1:100 (G). Odd-numbered lanes show the samples without PMCA. Even-numbered lanes were subjected to PMCA. The PrP detection antibody was 3F4. PrP^c^, normal cellular prion protein; CWD, chronic wasting disease; PMCA, protein misfolding cyclic amplification; vCJD variant Creutzfeldt-Jakob disease.

Dilutions of CWD brain homogenate in substrates prepared from human brain, transgenic mouse brain, and 239F cells expressing human PrP (129M or 129V) were compared for their ability to support amplification. Irrespective of origin, all 3 *PRNP* 129M-containing substrates supported amplification, albeit with slightly different efficiencies (Figure 3, panels A, B, C). All three *PRNP* 129V-containing substrates also supported amplification (Figure 3, panels D, E, F), although the level of amplification was lower than for the 129M equivalent. PMCA reactions using vCJD brain homogenate as a seed were conducted by using these same substrates and are shown for comparison (Figure 3, panel G).

**Figure 4:**
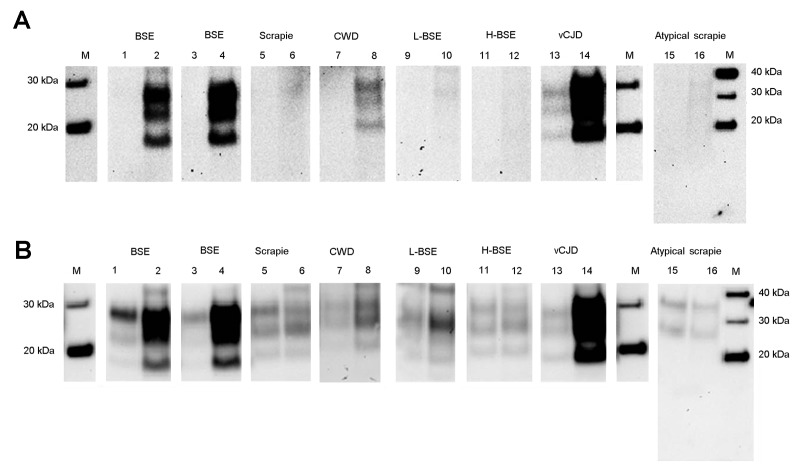
Relative conversion efficiency of human PrP (129M) by different animal prion disease samples. Brain homogenates from animal prion diseases were seeded at different volumes adjusted to give roughly equivalent amounts of seeding PrP^res^ and amplified by using PrP 129M-containing human brain substrate. Human PrP^res^ formation was detected by the 3F4 antibody (A) and seed and newly formed PrP^res^ detected using the 6H4 antibody (B). PrP, protein prion; PrP^res^, protease-resistant PrP; M, molecular marker; BSE, bovine spongiform encephalopathy; CWD, chronic wasting disease; L-BSE, L-type BSE; H-BSE, H-type BSE; vCJD variant Creutzfeldt-Jakob disease; M, molecular marker.

Next, we estimated the relative PrP^res^ amount in each sample by densitometry and adjusted the volume of 10% brain homogenate from the different animal prion diseases used in the PMCA reaction to give roughly equivalent amounts of PrP^res^ seed in each reaction (Figure 4). The amount of PrP^res^ in the atypical scrapie specimen was so low that a maximum volume of homogenate was used. The results of the Western blot analysis of these seed PrP^res^ normalized PMCA reactions, using antibody 3F4, confirmed that amplification efficiency was a function of seed/substrate compatibility and not simply PrP^res^ abundance.

**Figure 5:**
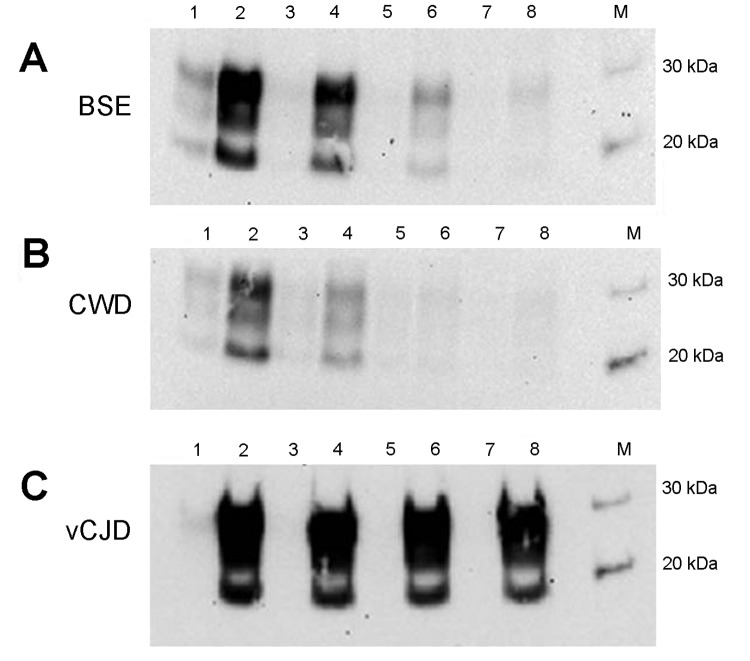
Properties of C-BSE, CWD, and vCJD amplification products in a second round of PMCA. Hu-C-BSE, hu-vCJD, and hu-CWD (from a previous round of PMCA) were supplemented with fresh human brain homogenate and subjected to a second round of PMCA. The reactions were normalized by PrP^res^ level and the product diluted (1:3, 1:6, 1:12, 1:24) in fresh human brain homogenate (*PRNP* codon 129MM) before PMCA. Odd numbers correspond to samples without PMCA; even numbers correspond to the reactions after PMCA (A, B, and C). The PrP detection antibody was 3F4. C-BSE, C-type bovine spongiform encephalopathy; CWD, chronic wasting disease; vCJD variant Creutzfeldt-Jakob disease; PMCA, protein misfolding cyclic amplification; hu, human; PrP^res^, protease-resistant prion protein; M, molecular marker.

C-type BSE, vCJD, and CWD amplification products were normalized and diluted (1:3, 1:6, 1:12, 1:24) in fresh human brain tissue homogenate (129MM) and subjected to a second round of PMCA. The CWD and C-type BSE PMCA reaction products retained their ability to convert further human 129M PrP, albeit at a lower efficiency than vCJD (Figure 5).

**Figure 6:**
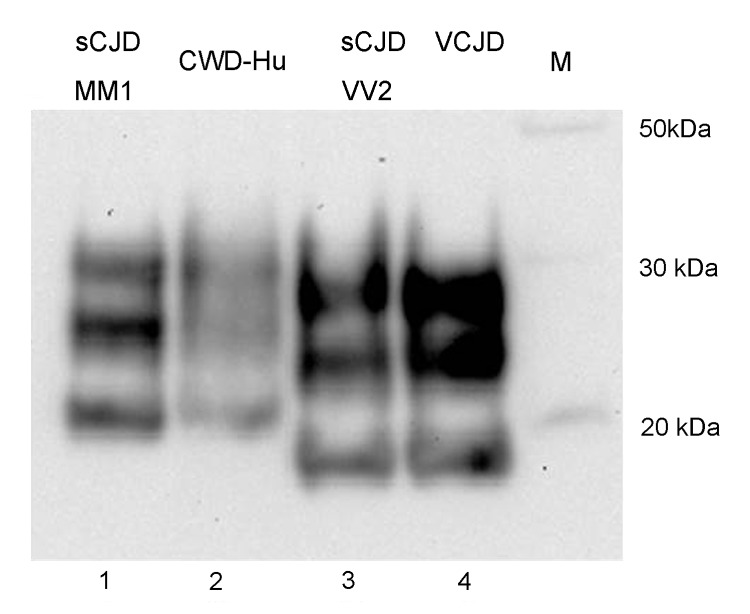
PrP^res^ typing of the CWD amplification product. The CWD PMCA product derived from amplification in a human brain homogenate substrate (*PRNP* codon 129MM) was compared by Western blotting with PrP^res^ from human brain samples from cases of sCJD of the MM1 subtype, sCJD of the VV2 subtype, and variant CJD. The PrP detection antibody was 3F4. PrP^res^, protease-resistant prion protein; CWD, chronic wasting disease; PMCA, protein misfolding cyclic amplification; sCJD sporadic Creutzfeldt-Jakob disease; vCJD, variant CJD; hu, human; M, molecular marker.

Western blot analysis of PrP^res^ produced by a PMCA reaction using human brain homogenate (129MM) seeded with CWD brain homogenate (Figure 6, lane 2) showed that this PrP^res^ shared the mobility and general glycosylation profile of type 1 PrP^res^ from sCJD brain (MM1 subtype) (Figure 6, lane 1). It was distinct from that of type 2 PrP^res^ characteristic of sCJD (VV2 subtype) and vCJD (Figure 6, lanes 3 and 4, respectively).

## Discussion

Multiple factors govern the transmission of prions in experimental settings. In addition to infectious dose and route, a species or transmission barrier phenomenon is well recognized. Within the theoretical confines of the prion hypothesis, the most obvious basis of a species barrier effect would be dissimilarity in *PRNP* sequence between the infectious source and the exposed individual. However, *PRNP* sequence similarity alone does not seem to accurately predict whether prions are transmissible between species, perhaps because interactions between PrP^C^ and PrP^Sc^ occur as native PrP^C^ and misfolded and aggregated PrP^Sc^ conformers. The possible effects of species-specific sequence difference on PrP^C^ folding are not well understood. Neither is the secondary and higher order structure of PrP^Sc^_,_ except for clear evidence that different prion strains are associated with different PrP^Sc^ conformers and glycotypes (reviewed in *4**,**18*) and that these might exist as a quasispecies or molecular cloud (*19*). Under such a scenario molecular compatibility might be difficult to predict.

To isolate and study molecular effects, we have previously conducted cell-free PrP conversion experiments by PMCA using homogenates of bovine and ovine prion disease brain samples to seed brain homogenates containing human PrP, assessing the extent of conversion by detection of human PrP^res^. These studies showed that samples of C-type BSE (which is a known human pathogen and the cause of vCJD) efficiently converted human PrP, with a codon 129 preference similar to that of vCJD (MM>MV>VV), whereas samples of classical scrapie (which is not thought to be a human pathogen) failed to convert human PrP to a measurable extent. Equally importantly, a sheep BSE isolate resembled C-type BSE and vCJD in its ability to convert human PrP, thus underscoring influence of strain over sequence similarity in determining what might be termed a molecular transmission barrier (*10*).

Here we applied the same approach to a series of animal prion diseases whose risk to human health is poorly characterized. Our results show that under the PMCA conditions used, L-type BSE, H-type BSE, and atypical scrapie isolates fail to produce detectable human PrP^res^. The CWD isolate used converted human PrP^C^, albeit less efficiently than C-type BSE. This observation remained true whether the input animal prion disease brain homogenate was normalized by tissue weight or by PrP^res^ abundance and whether the PMCA substrate was from human brain, *PRNP* humanized murine brain, or a human-derived and human PrP^C^ overexpressing cell line. The conversion of human PrP^C^ by CWD brain homogenate in PMCA reactions was less efficient when the amino acid at position 129 was valine rather than methionine. Furthermore, the form of human PrP^res^ produced in this in vitro assay when seeded with CWD, resembles that found in the most common human prion disease, namely sCJD of the MM1 subtype.

Previous attempts to determine the transmissibility of these prion diseases to humans and thus assess their zoonotic potential have used experimental challenge of nonhuman primates, humanized PrP transgenic mice, and cell-free assays with sometimes conflicting results. Successful transmission of CWD and L-BSE to certain nonhuman primates has been reported: L-type BSE showing a different pathologic profile and a shorter incubation period than C-type BSE (*20*–*23*). However, Kong et al. (*24*) reported that CWD failed to transmit to humanized PrP 129M overexpressing mice inoculated with an elk brain homogenate. In contrast, Beringue et al. (*25*) reported that humanized PrP 129M overexpressing mice were susceptible to L-type BSE and suggested that L-type BSE was more virulent than C-type BSE and presented a zoonotic risk. H-type BSE reportedly failed to transmit to these same mice. Sandberg et al. (*26*) and Tamgüney et al. (*27*) confirmed the previous report of Kong et al. that CWD fails to transmit to transgenic mice, irrespective of whether 1) the mice expressed bovine, ovine, or human PrP; 2) the mice expressed the human 129M or 129V PrP allelic variants; or 3) the CWD isolates were from mule deer, elk, or white-tailed deer.

Cell-free approaches to modeling human susceptibility to animal prion prion diseases also have been published (*8*,*10*,*28*–*31*). Raymond et al. (*28*) compared the ability of CWD, C-type BSE, sheep scrapie, and CJD brain homogenates to convert human PrP^C^ metabolically labeled and purified from transfected cells. These experiments obtained limited conversion of human PrP^C^ by CWD, C-type BSE, and scrapie. In contrast to our study, this early cell-free system failed to distinguish between scrapie and C-type BSE in their ability to convert human PrP^C^; however, it indicated a substantial molecular barrier to conversion of human PrP^C^ by CWD PrP^Sc^ (*28*,*29*), which agrees with this report. Kurt et al. (*31*) reported that PMCA using human PrP^C^ overexpressing transgenic mice brain (both 129M and 129V lines) as substrate failed to support amplification when seeded with CWD cervine brain homogenate. Cervidized *Prnp* transgenic mouse brain homogenate can support CWD prion replication (*32*), and extensive in vitro conditioning of a CWD isolate by PMCA in a cervidized substrate (or passage in cervidized mice) was sufficient to overcome the barrier and enable efficient in vitro amplification in a humanized transgenic mouse substrate (*33*). Direct comparison of these studies is made difficult by the differences in approach (in vivo vs. in vitro), the different transgenic constructs used, and the technical details of the cell-free conversion assays undertaken (Table). An additional possibly significant difference between these studies is the nature of the CWD isolate used. CWD affects different deer species (some of which show allelic variation in their *Prnp* sequence), but CWD also occurs as different biologic strains of agent (*34*–*36*). Different strains of CWD may have a role in determining transmissibility and conversion efficiency. Recently, Meyerett et al. (*37*) reported the in vitro strain adaptation of a CWD isolate by serial PMCA, similar to that produced by in vivo subpassage.

**Table:** Comparison of the outcomes of experimental transmission and in vitro conversion studies of chronic wasting disease in human, humanized, and nonhuman primate model systems*

*Donor animal inoculum*	*In vivo*				*In vitro*				*Ref*
	Species inoculated	Animal (expression levels)/codon 129	Transmission		PrP source (expression levels)/codon 129	Method		Conversion	
Mule deer, white-tailed deer, and elk					Cells 129M and 129V	C-FA		Pos	(28,29)
Elk	Humanized PrP transgenic mice	Tg-40 (1×)/129MM	Neg						(*24*)
		Tg-1 (2×)/129MM	Neg						
Mule deer	Squirrel monkeys	*Saimiri sciureus* (1×)	Pos						(*20*)
Mule deer, white-tailed deer, and elk	Humanized PrP transgenic mice	Tg(HuPrP)440 (2×)	Neg						(*27*)
Mule deer, white-tailed deer, and elk	Squirrel monkeys	*Saimiri sciureus* (1×)	Pos						(*22*)
	Cynomolgus macaques	*Macaca fascicularis* (1×)	Neg						
Mule deer and white-tailed deer					Tg-6816 (16×)/129M	PMCA		Neg	(*31*)
					Tg-7823 (5×)/129V	PMCA		Neg	
Mule deer	Humanized PrP transgenic mice	Tg-45 (4×)/129MM	Neg						(*26*)
		Tg-35 (2×)/129M	Neg						
		Tg-152 (6×)/129VV	Neg						
White-tailed deer	Humanized PrP transgenic mice	HuMM (1×)/129MM	Neg						(*11*)
		HuVV (1×)/129VV	Neg						
Mule deer					Tg-440 (2×)/129MM		PMCA	Pos (after in vitro conditioning)	(*33*)
Elk					Human brain (1×)/129MM		PMCA	Pos	This article
					Human brain (1×)/129VV		PMCA	Pos	
					HuMM (1×)/129MM		PMCA	Pos	
					HuVV(1×)/129VV		PMCA	Pos	
					293F cell line(4×)/129M		PMCA	Pos	
					293F cell line(2×)/129V		PMCA	Pos	

*Numbers in parentheses denote the stated expression levels of PrP^c^ into the animal species and cell lines used. PrP, prion protein; C-FA, cell-free assay; pos, positive; neg, negative; PrP^c^, host’s normal cellular PrP. Blank cells indicate no reported data.

The most directly comparable in vivo study to that reported here is Wilson et al. (*11*), in which a similar series of atypical animal prion diseases were used to challenge transgenic mice expressing physiologic levels of human PrP^C^. Atypical scrapie; C-, H-, and L-type BSE; and CWD all failed to produce disease (or signs of infection) on first passage in these mice (*11*). The use of different animal prion disease isolates (and possibly differing species and strains of CWD) might explain this discrepancy; however, a more fundamental difference might be that the in vivo and in vitro model systems assess different aspects of the agent and its replication. The in vivo model is undoubtedly more complex and arguably more physiologically relevant, and the readout is disease; however, it remains disease in a mouse, in which the *PRNP* sequence alone is human. The in vitro cell-free model does not assess disease as such, only the compatibility of particular combinations of seed and substrate homogenates (some of which, in these examples, were entirely of human origin) to produce PrP^res^. Differences between the in vivo and in vitro models are exemplified by the comparison of C-type BSE, and vCJD. Both amplify well in PMCA using humanized (129MM) brain homogenate as a substrate (*10*), whereas intracranial inoculation of C-type BSE into humanized (129MM) mice fails to produce disease (*12*), unless first experimentally transmitted to sheep or goats (*13*,*38*,*39*).

The interpretation of different amplification efficiencies as a semiquantitative measure of relative risk is tempting but is probably premature and almost certainly an oversimplification. The testing of more isolates, especially of CWD in deer and elk, is advisable before any firm conclusions can be drawn. Additionally, possible strain-specific effects on amplification efficiency by the precise PMCA experimental conditions are difficult to discount and might complicate interpretation. The relative amplification efficiencies of C-, H-, and L-type BSE might differ intrinsically because certain strains of sheep scrapie appear to, even when amplified in homologous sheep substrates (*40*). However, we can say with confidence that under the conditions used here, none of the animal isolates tested were as efficient as C-type BSE in converting human PrP^C^, which is reassuring. Less reassuring is the finding that there is no absolute barrier to the conversion of human PrP^C^ by CWD prions in a protocol using a single round of PMCA and an entirely human substrate prepared from the target organ of prion diseases, the brain.
